# The Food Resources of Russia

**Published:** 1855-12

**Authors:** 


### JOURNAL OF PUBLIC HEALTH.

DECEMBER 1855.

### THE FOOD RESOURCES OF RUSSIA.

An important work, lately issued by the Board of Trade, contains some curious information on the internal resources of the Russian empire. The volume itself is as uninviting as one of Franklin's sawdust dumplings. It presents a little sea of blue without, and of double black within. To speak of its *alpha* or its *omega* would be a perversion of terms, for it is made up of figures from beginning to end. Turn over any page you may, the effect is the same—columns upon columns of dismal numbers; columns short and plethoric; columns long and thin; columns with a broad base and a tapering head, that need not be spoken of; columns with hour-glass waists; columns in every unimaginable form of distortion;—in short, columns of as many varieties and sizes as there are varieties of men, women, and manners.

Always excepting "Bradshaw", there are no works more difficult to peruse than these governmental records. Hence, doubtless, the reason why so few people read, mark, learn, and inwardly digest them. Whether the indefatigable authors of these works do ever themselves deduce any simpler conclusions from them than those which are expressed in "sum totals", or whether our ruling legislators make any profound use of the information so laboriously gathered, are questions which it might be difficult to answer. It will be sufficient, on this occasion, to dig out a few important facts from the first section of the volume in hand, to place these facts before the public in a readable form, and, literally speaking, in our mother tongue.

This first section includes statistics bearing upon the population, the extent of cultivation, soil, and the food resources of Russia, exclusive of Poland and Finland.

Before the commencement of the war, viz. in 1849, there were 53,137,150 inhabitants in this great northern empire. As is not uncommon, the ladies took the lead in the matter of numbers. The excess of females over males was 412,889; a difference which by this time the war must have considerably widened. This population altogether possessed, at the period spoken of, a surface of country embracing 1,675,492,948 acres of land; which, to the extent of rather more than a fifth part, was under cultivation. About 24,000,000 acres were private domains; 218,387,516 acres were devoted exclusively to arable purposes; 107,971,138 acres were pasture land; 393,277,413 acres were covered with forest wood; and 932,052,138 acres were waste. Deducting the waste and forest lands from the whole, there were thus left 350,163,397 acres of cultivated soil; which, on an uniform rate of distribution to population, would have given a proportion of more than six acres and a half to each one of the 53,000,000 of inhabitants.

On the cultivated parts, in the year 1849, a year of average produce, there were sown 45,592,043 quarters of corn, including rye, wheat, and other kinds of grain. These yielded 165,283,425 quarters. Of this produce, 67,410,156 quarters were wheat and rye, thus yielding an average of about one and one-twelfth of a quarter of what is called in Russia "bread corn" to each inhabitant, supposing an equal division. Of potatoes, there were sown 4,065,825 quarters, giving a produce of 12,752,573 quarters, or an average of a fourth part of a quarter to each person.

The number of animals used commonly for food included a total of 66,481,435: viz. of horned cattle, 21,228,240; of sheep, 35,335,663; of swine, 8,862,410; and of goats, 1,055,122. The average proportion of animal food to each person cannot be deduced. For the formation of sugar from beet, there were 307 manufactories; and 95,164 acres of land were under beet cultivation. The amount of sugar produced in the whole empire, including Poland, was 290,238 cwts.

The articles of food imported into the empire for home consumption were, in 1851, coffee, 73,544 cwts.; raw sugar, 588,175 cwts.; refined sugar (in 1848), 112,810 cwts.; wines and spirits, an amount producing in money £1,168,106; and olive oil, 8,819 tuns. As further articles of importation, there were also tea, fruit, spices, cocoa, fish, salt, tobacco, and porter. Among the exports in the year 1851, were wheat, 1,738,905 quarters; rye, 620,867 quarters; oats, 490,435 quarters; and barley, 205,914 quarters: the total amount of corn exported being 3,056.121 quarters.

These statistics, bearing on the food resources of Russia, are compiled from official returns, and afford an accurate view of the condition of our enemy's grand provision stores previously to the war.

It may be inferred that, at the period when these facts were chronicled, the produce of the empire itself was sufficient to supply all the inhabitants with those elements of food which are really essential to life. There was a sufficiency of corn, and possibly of animal food; but a comparative deficiency of potatoes. The chief articles of importation were luxuries, as wines and spirits, fruit, tea, and coffee. It must be remembered, also, that the masses of the Russian population, as they have fewer and simpler requirements than those of Great Britain, consume much less of the more highly nitrogenised varieties of sustenance. The whole produce of the land is moreover in the hands of the higher classes; and, the serf having to look to the master for the wherewithal he may be filled, the distribution of food amongst the lower classes is possibly conducted on a more uniform scale than in countries where men, in proportion to their means, buy and eat what they choose.

The practical point to be most considered at present is, whether Russia can, in the face of the war, continue to make her extensive soil still sufficiently productive to maintain her children in full physical health and vigour. Great as the losses of the Russian army have been, these losses can make but little difference to a giant population of 53,000,000. The loss of consumers thus occasioned cannot, in any way, compensate for the increase of food substance required by two or three marching and fighting armies, to say nothing of the absolute destruction of provision-stores, and the injury done to agriculture by the removal of large masses of working men from the ploughshare to the sword.

With these facts, it is not difficult to foresee that, unless large supplies of food are regularly imported into Russia, (a proceeding which is next to impossible under the existing pressure), the effect of a very few years, or even months, of continued warfare must inevitably destroy her independence in the matter of food supply for her people. It has been seen, it is true, that the Russian can possibly make a little food go a greater way than most of his world contemporaries. But economy, though it may ward off for a time a serious catastrophe, cannot create new resources; and if the war continue long under conditions as unfavourable to Russia as the present, the end must be a general famine and its inevitable sequelæ, increasing national depression, loss of physical force, and the general outbreak of those specific pestilences which, by virtue of fixed laws, find ever an origin and rapid development in the homes of starving populations.

---

### THE MEDICAL POLICE OF LONDON.

